# The Impact of the Food Labeling and Other Factors on Consumer Preferences Using Discrete Choice Modeling—The Example of Traditional Pork Sausage

**DOI:** 10.3390/nu12061768

**Published:** 2020-06-12

**Authors:** Péter Czine, Áron Török, Károly Pető, Péter Horváth, Péter Balogh

**Affiliations:** 1 Department of Statistics and Methodology, Institute of Statistics and Methodology, Faculty of Economics and Business, University of Debrecen, 4032 Debrecen, Hungary; czine.peter@econ.unideb.hu (P.C.); balogh.peter@econ.unideb.hu (P.B.); 2 Department of Agricultural Economics and Rural Development, Institute for the Development of Enterprises, Corvinus University of Budapest, 1093 Budapest, Hungary; 3 Department of Rural Development and Regional Economics, Institute of Rural Development, Regional Management and Tourism Management, Faculty of Economics and Business, University of Debrecen, 4032 Debrecen, Hungary; peto.karoly@econ.unideb.hu (K.P.); horvath.peter@econ.unideb.hu (P.H.)

**Keywords:** food labeling, latent class modeling, traditional meat product, mangalica sausage

## Abstract

In our study, we examined whether product characteristics indicated by food labels matter in purchasing decisions for sausage made from traditional Hungarian mangalica pork; and how much consumers are willing to pay for them. On the other hand, we also tried to measure whether any changes in consumers’ preferences occurred in recent years. Two product characteristics (label of origin and different mangalica meat content) and two other factors (place of purchase and price) are examined in a discrete choice experiment based on stated preference data. According to our expectations, government-funded consumer campaigns in recent years have had an impact on consumers purchase of this traditional product, and they pay more attention to food labels, which can also be influenced by sociodemographic characteristics. Our results have been compared to a previous choice-model based research, investigating consumers’ attitude towards similar mangalica pork products. Three different types of models (multinomial logit, random parameter logit, and latent class) are employed, from which two types of models account for the heterogeneity in preferences. Based on the results, it can be concluded that the advertisements promoting traditional meat consumption had only a partial effect on consumer attitudes. Consumers clearly prefer the label of origin indicating meat from registered animals and purchasing on the farmers’ market, but according to the indication of the different mangalica meat content in the product, we have already reached conflicting results. Three consumer segments were identified: “price sensitive, loyal to label, label neutral” based on latent class model estimates.

## 1. Introduction 

Research in consumer preferences has always been a central topic of economics, especially in microeconomics, which is highly related to the field of marketing. This relationship is mainly focused on answering questions on the preferences of members of groups with different characteristics and how they make their purchasing decisions [1]. Food labels may represent a marketing tool and may influence consumers’ perception of food quality [2].

In Hungary, there is growing interest and increasing consumer demand for high-quality and healthy local products [3,4]. The intensification of local food products’ production would contribute improving ecosystems and would help towards improving the economic situations of farmers, who form the basis of local development, and are directly related to the achievement of United Nations’ second, 12th, and 15th sustainable development goals [5].

Habits and traditions linked to countries and territories have always played an important role in food choices; therefore, it is often investigated by agricultural marketing. In six European regions, Guerrero et al. [6] examined what the word “traditional” means for consumers in terms of food. They found that consumers from southern Europe mostly associate to heritage, culture, or history, while central and northern Europeans rather to convenience, health, or appropriateness. In contrast, Kühne et al. [7] tried to gauge how people perceive traditional product innovation. Consumers’ reactions indicated openness towards innovations in this product category, however, with preserving the traditional character as a prerequisite for innovations. Pieniak et al. [8] analyzed the relationship between traditional food consumption and food choice motifs and found that the familiarity with the product and the natural contents are positively associated with the attitudes towards traditional foods. Chrysochou et al. [9] examined the role of quality labels as a driver of consumer loyalty in traditional foods. Based on French scanner data, results showed that standalone labels assuring the designation of origin are less important than brands accompanied with such origin labels. 

Mangalica pig is considered a traditional Hungarian breed with Serbian origin, emerged in the Carpathian basin [10], widely used in Hungary from the middle of the 19th century, mainly due to its undemanding nature and excellent lard producing capacity [11]. However, due to changing consumer habits requiring a less fatty diet, the number of registered sows fell from 18,000 heads in 1955 to 243 in ten years, and in another five years, to a few dozens of animals [12]. The breed was rescued after the political change in Hungary, in the mid-1990s when the Hungarian National Association of Mangalica Breeders was established, which certifies the pigs and officially guarantees the origin of genuine mangalica products. In 2004, the Hungarian Parliament claimed mangalica a national treasure representing a high genetic value. Since then, several governmental initiatives took place to strengthen its position, among others marketing campaigns, to promote mangalica food products. Therefore, in recent years, the number of registered sows has increased from 7327 to 10,050 [13]. There has been a continuous growth in the economic importance of the breed due to the increase in demand both in the domestic and export market: Hungarian consumers mainly seek for sausage made from mangalica meat, while a Spanish serrano ham producer sources mangalica meat, producing top quality products [10,11].

Individuals make decisions daily, choose from alternatives; therefore, it is exciting how and based on what they do this [14,15]. The full explanation is probably impossible to find because decision-making is characterized by a high degree of variability and heterogeneity due to several uncertain factors that are mostly difficult or impossible to measure [16]. The purpose of examining the decision-making process is to identify as much information as possible to facilitate a fuller, more detailed understanding. Closer analysis of the chosen alternative (product or service) can provide significant help in this regard [17]. The term preference is rarely used in everyday life, but it is a regular part of life. These are mostly based on the attributes of the product/service (e.g., label, taste, price, etc.), which can be positive or negative (avoiding something). So it can also be said that choices are made within specific attributes of alternatives in the decision-making process. However, it is essential to know which characteristics (e.g., label, taste) are relevant and how important they are for decision-makers. If one aspect is considered important, this factor will be the basis for comparing alternatives. However, if several aspects are decisive, combinations thereof are compared [18]. By assigning a numerical value to them, we can find out the satisfaction or utility level of the consumers. An important consideration when examining preferences is to take proper account of the various limitations. These may include income limits (resulting from not necessarily having the amount of money at which the alternative could be obtained) and technological limitations (resulting from the fact that the product/service requested is no longer or not yet available under current market conditions) [19].

In the literature, there are two main methods identified to assess consumers’ choices. The revealed methods observe the behavior of individuals in real-world market situations. In contrast, the stated methods confront consumers with a hypothetical situation to evaluate alternatives of labels that are not yet available in the real market environment [20,21,22]. The latter can be particularly useful when it is about whether certain products/services with different labels should be introduced to the market [23,24]. It is important to mention that there are many investigations with a combination of revealed and stated data together, with labels aimed at gaining more relevant information through the results [25,26]. This approach is often used in the process of evaluating consumer preferences, also known as discrete choice experiment [27,28,29]. 

The discrete choice experiment is based on the utility-maximizing behavior of individuals. It means that the element with the highest utility value is always selected from a deciding set. Furthermore, according to the theory of characteristics, the utility of products/services derives from their attributes. In addition, it assumes a discrete choice situation (only one element is selected from the choice set) [30]. Finally, the utility function is broken down into a systematic and a random part [31].

The method is increasingly appearing in the field of agricultural marketing, where the focus is on examining the expected attributes of different foods (e.g., label of origin, price, fat, or meat content) and the willingness to pay for product attributes (e.g., how much they are willing to pay for a label indicating 25% higher meat content) [31,32,33,34,35]. 

Therefore, in this paper, we would like to answer several questions. First, which kind of characteristics of the traditional Hungarian mangalica sausage matters for the consumers. Second, what is their willingness to pay for these attributes, third can we distinguish different traditional product consumer segments using the latent class (LC) model and fourth, can we observe any kind of change in consumers’ preference for this traditional product in the recent years, mainly due to the government-funded promotions. 

## 2. Literature Review 

Among foods, the traditional and regional meat products have always played a prominent role, especially if they are certified (e.g., geographical indication, country of origin, etc.). In the regions related to production, usually, local consumers prefer traditional meat products, and it is often accompanied by a higher willingness to pay [36]. On average, meat products have the highest price premium among foods with geographical indication in the European Union [37]. In the case of raw meat, the literature is mainly focusing on beef (e.g., [38,39,40,41]) and lamb (e.g., [42,43,44,45,46]), indicating that consumers are positive and willing to pay more for local and traditional products or with origin with high reputation. In the case of pork meat, for hams, in particular, several studies indicated that consumers are particularly fond of these traditional products and are ready to pay more for them (e.g., [47,48,49]). However, certifications and trademarks are only relevant up to a certain level of quality and in the very premium segment, other product attributes matter more [29]. It is also important to point out that for meat products with a high reputation, consumers living near the place of production tend to be willing to pay a lower premium than those from remote areas [50].

Many research focused on consumers’ attitudes toward pork meat. Among others, Balcombe et al. [51] examined consumer preferences for different meat products with special emphasis on country of origin, using a discrete choice experiment. Based on their results, the country of origin information is positively assessed for all food products. However, it was already considered less important compared to other food product attributes. Shan et al. [52] investigated consumer ratings for processed meat products. The study identified consumer purchase intentions and quality perceptions of basic meat products. These included the price, base meat product, and healthy ingredients. Di Vita et al. [53] emphasized that the price is one of the most important factors in determining the quality level of the salami investigated. Besides, the authors concluded that certain socio-economic segments of consumers show a significant willingness to pay extra for low salt content and nitrate-free salami. Ngapo [54] explored consumer preferences in the direction of pork ribs in five Canadian provinces. According to his findings, fat cover and slight color were among the factors most influencing consumer choices. Špička et al. [55] investigated consumer behavior in the Czech pork retail market. They identified that the Czech consumers preferred packaged meat or counter sales over sourcing from a butcher. Furthermore, the relatively high proportion of low education consumers and one-person households do not favor quality; people with lower education are more price-sensitive than those with higher education; older consumers prefer price to quality; in general, the majority of consumers prefer domestic pork by origin. Font-i-Furnols and Guerrero [56] examined consumer behavior and perception of meat and meat products. Based on their results, consumer behavior is influenced by several factors related to meat and meat products, so consumer preferences are heterogeneous. Effective information strategies (labels in particular) can help to promote a better understanding of the process. Xazela et al. [57] examined perceptions of rural consumers regarding meat consumption. They assessed that the main place of purchase was the supermarket, which was rated the most hygienic and with a fresh supply. They further conclude that consumers’ perceptions of meat quality are also influenced by their income and cultural background. Merlino et al. [58] examined the behavior of households concerning meat consumption, with a particular focus on households with and without children. The results showed that weekly meat consumption was higher in households without children. Both groups preferred sourcing from the butcher, followed by supermarkets. Kallas et al. [59] attempted to assess consumer preferences for traditional and innovative pig products. Their results showed that preferences are heterogeneous among the countries included, moreover eating experiences have a significant effect on preferences. Kallas et al. [60] used a non-hypothetical discrete choice experiment with a hedonic evaluation to find out the importance of food neophobia in consumer purchases and to calculate willingness to pay before and after tasting the products. Their results showed that traditional pork products preventing cardiovascular disease achieved higher purchase intention and willingness to pay (WTP) than expected. However, after tasting, consumers showed a lower WTP for all innovative traditional pork products. Food neophobia was closely related to WTP prior to hedonic evaluation. Candek-Potokar et al. [61] examined the sustainability of local pig breeds by establishing a collaborative trademark. Among others, they suggested to have a label attracting end-users (farmers), breeders associations, and meat processors to sufficiently support conserving local pig breeds.

Balogh et al. [62] also focused on a traditional meat product: salami made of mangalica pork. The survey they employed was conducted between August and October 2012, in the Northern Great Plain region of Hungary, using a discrete choice experiment with 309 participants. The main findings of the research were that consumers prefer a salami product made entirely of mangalica meat and sourced from a butcher. During the years after the experiment (between 2012 and 2018), the Hungarian government placed great emphasis on encouraging the consumption of locally produced (labeled) traditional products, including mangalica meat, from reliable sources. In 2019, with the support of the Ministry of Agriculture, the incentive program of the Agricultural Marketing Center was launched (including so-called “Mangalica Festivals”, tastings, chef competition, conference held in several cities), which was explicitly aimed at promoting mangalica meat and encouraging its consumption [63]. In the light of the above, our research tries to find out whether there has been a change in consumer preferences for labeled mangalica meat (represented by a sausage product in the present study) compared to the results of this previous research. 

Against this background, we tried to investigate four different aspects of consumers’ attitude towards mangalica sausage.

First, what are the product attributes that matter for the consumers?

Second, what are their willingness to pay for these attributes?

Third, can we distinguish different traditional product consumer segments using the LC model?

Finally, is there any change in consumer preference as a result of the marketing campaigns financed by the Hungarian government? Here, as a benchmark, we used the results of similar research conducted in 2012 [63]. 

## 3. Materials and Methods 

Before the survey, two focus group interviews were conducted (with six regular consumers per group) in October 2019 to determine the product attributes to be included. We identified four attributes including price (representing four typical price levels for similar products available in the region of the research *and our price levels are the same as the previous research of Balogh et al. (2016) used, as the price of mangalica sausage and salami did not change significantly in the recent years and to get more comparable results*), mangalica meat content (the proportion of mangalica in the product), label of origin (official label of the breeding association certifying that the product was made from registered mangalica pigs raised in a registered barn), and the place of the purchase (the type of sales channel) were included, similar to the research of Balogh, Békési, Gorton, Popp, and Lengyel [62]. Subsequently, we determined their attribute levels, all referring to the real market conditions (Table 1). 

We used so-called “design” coding for categorical variables (here, there was a difference compared with Balogh et al. [62] because they used “effect” coding in their models run in STATA software). In all cases, the same levels were the base levels during the model estimations (50% meat content, No NAMB label of origin, Farmers’ market). To select the experimental design, we first estimated how many decision situations the full factorial design would include in the experiment. Based on the selected attributes and their levels, this number became too high (72), so we used a partial D-efficient design (to reduce the number of product combinations while minimizing the number of D-errors), through the use of Ngene 1.2. software in the final questionnaire, with eight decision situations (with three alternatives per situation, each with a “no answer” option) [64]. See Table 2, for example.

In addition to the decision situations, the final questionnaire included additional questions on purchasing and consumption habits and the sociodemographic characteristics of the respondents. Before the survey, we did a pilot survey to get feedback on difficult-to-understand parts. 

Our research was carried out in the three most populous cities of the Northern Great Plain region (Nyíregyháza, Debrecen, Szolnok), where 26% of the registered Hungarian mangalica sows are kept, and the headquarter of the National Association of Mangalica Breeders (NAMB) is also located. Moreover, the most substantial volume of mangalica products is purchased in this region. In the three selected cities, respondents were reached in front of the stores of the Tesco hypermarkets, between December 2019 and February 2020. The sample contains 477 persons (155–165–157 answers, respectively), detailed in Table 3. During the data collection, great emphasis was placed on ensuring representativeness for several sociodemographic variables through quota sampling. We have successfully achieved this by gender, age, and place of residence [65,66].

The dataset was processed via the R: Apollo 0.0.6 software extension [67,68]. In the following, we describe the characteristics of the model specifications used in estimates. 

### 3.1. Multinomial Logit Model (MNL)

The widely used multinomial logit model is related to McFadden and Zarembka [69], which has the advantage that estimates can be easily interpreted. It is based on the theory of random utility; it assumes the utility-maximizing behavior of individuals. For the model, the systematic and random part of utility can be written according to Equation (1):

(1) $$U_{n,i}=\beta X_{n,i}+{Ɛ}_{n,i},$$

where *U* denotes the total utility, *βX* the systematic part, *Ɛ* the random part, *n* the respondent, and *i* denotes the alternative. 

The probability of choosing alternative *i* is given by Equation (2):

(2) $$Prob_{n,i}=\frac{\exp\sum_{k=1}^{K}\beta_{k}X_{n,i,k}}{\sum_{i=1}^{I}\exp\sum_{k=1}^{K}\beta_{k}X_{n,i,k}},$$

where *k* is the product/service attribute, *X* is the variable, *β* is the coefficient value for that variable.

One of the drawbacks of the model is that it is unable to capture the heterogeneity of individual tastes and assumes the independence of irrelevant alternatives. As a result, more complex models are often used to analyze discrete choice experiments [70]. 

### 3.2. Random Parameter Logit Model (RPL)

The major advantage of the random parameter logit model is its ability to capture preference heterogeneity. This is reached by allowing the coefficients of *β* to be randomized among the respondents, along with a particular pre-selected distribution by the researcher. At the same time, their parameters (mean, standard deviation) can also be set up [71]. The other great advantage is to allow a flexible variance-covariance structure for the random term, thus resolving the restrictive assumption known as the independence of irrelevant alternatives [72,73]. In the model, the systematic part of the utility can be decomposed by Equation (3):

(3) $$V_{n,i}=\left({\bar{\beta }}^{\prime }+\eta_{n}{}^{\prime }\right)\;X_{n,i},$$

where $\bar{\beta }$ is the mean value, and $\eta_{n}$ a person-specific difference. 

The probability of choice (for alternative *i* by person *n* in decision situation *t*) can be described by Equation (4):

(4) $$Prob_{i,n,t}=\frac{\exp\left(\alpha^{\prime }+\beta^{\prime }X_{i,n,t}+\varphi^{\prime }F_{i,n,t}\right)}{\sum_{j=1}^{J}\exp\left(\alpha^{\prime }+\beta^{\prime }X_{j,n,t}+\varphi^{\prime }F_{j,n,t}\right)},$$

where $\alpha^{\prime }$ is the alternative-specific constant value, $\beta^{\prime }$ is the random parameter, $\varphi^{\prime }$ is the fixed parameter, $X_{i,n,t}$ is the attribute variable for the alternative, and $F_{i,n,t}$ is the variable of personal characteristics [74].

### 3.3. Latent Class Model (LC)

An advantage of the latent class model is that it captures heterogeneity among individuals. This is reached by grouping individuals into distinct *Q* groups, which are distinct and have their own *β* parameter [75]. In the model, the probability of choosing alternative *i* by person *n* from class *q* can be described by Equation (5) [76,77]:

(5) $$Prob_{i,n│q}=\frac{\exp\left(\beta_{q}^{\prime }X_{i,n}\right)}{\sum_{i=1}^{I_{n}}\exp\left(\beta_{q}^{\prime }X_{i,n}\right)}\;q=1,\ldots ,Q.$$

One of the features of the latent class model is to estimate class probability values ($H_{n,q}$), which allows estimating the probability of individuals falling into different classes. Accordingly, the probability of choice changes to Equation (6):

(6) $$Prob_{i,n}=\overset{Q}{\underset{q=1}{\sum }}Prob_{i,n│q}H_{n,q}.$$

The limitations of the model most often refer to the ideal definition of the number of classes. Most decisions are made based on information criteria (Akaike information criterion (AIC), consistent Akaike information criterion (CAIC), (Bayesian information criterion (BIC) [78]. 

## 4. Results 

In the following, we intend to present estimates of the three models. Firstly, we use multinomial logit, then the random parameter logit models, and finally, we estimate latent class model in order to find whether we can distinguish different consumer segments based on their preferences for a food product [79,80]. The utility functions used for the different models, and how the willingness to pay (in case of MNL model) was calculated, are explained in Appendix A.

### 4.1. Importance of Different Product Attributes and Willingness to Pay among Mangalica Sausage Consumers

The results of our estimates based on MNL and RPL (where we used a special form utility function to make direct estimates for the willingness to pay [73]) specifications are presented in Table 4.

In the RPL model, all parameters were first randomized; however, we got significant standard deviation values only for the price, label of origin, and hyper-/supermarket variables. As a result, only these were randomized in the final specification (as shown in Table 4) [19]. We chose log-uniform distribution for the price, while we used normal distribution for the label of origin and hyper-/supermarket [81]. For the estimates, we used 500 so-called halton draws. 

Based on the results of the MNL model parameter estimates, it can be concluded that the opt-out alternative (no buying sausage) was preferred significantly less often than the alternative 1; as the price of the mangalica sausage increases, the level of consumer utility decreases; as the mangalica meat content increases, consumers sense of utility increases; the existence of label of origin has a positive impact on utility; they prefer purchasing on the ‘farmers’ market over butcher and much over the hyper-/supermarket. According to the significance values, all parameters in the model can be considered significant.

For the MNL model, calculations of willingness to pay were based on point estimate [19]. The results are shown in Table 5.

According to the WTP estimates, consumers would pay more for higher meat content, ca. 787 HUF more for products with 75%, and 954 HUF more with 100% mangalica meat content, compared to 50% meat content. In addition, they would pay about 2082 HUF extra for a labeled product certifying the origin. Finally, they would pay less outside of ‘farmers’ market, while results estimated a discount of 858 HUF at a butcher and 1139 HUF in a hyper-/supermarket. 

Comparing the results of MNL and RPL models, we can see that the RPL model specification shows a significantly better fit (Pseudo R^2^ in MNL: 0.1608; Pseudo R^2^ in RPL: 0.2634). Furthermore, significant standard deviation values are present for some attributes (price, label of origin, hyper-/supermarket), indicating that there is heterogeneity in preferences which the MNL model cannot handle. The significant ASC (opt-out) value of RPL model (−3.191) means that consumers preferred more alternative 1 over the no-choice alternative. According to the negative value of the price coefficient, consumer utility decreases when the price increases. There is a statistically significant standard deviation value for the price attribute, which means that there is heterogeneity in consumer preferences regarding price. There is a difference in mangalica meat content compared to the estimates of the MNL model. Consumers prefer more a 75% than a 100% mangalica meat content product compared to a 50% mangalica meat content product (they would pay about 895 HUF more for 75% and 862 HUF more for 100% mangalica meat content product, compared to 50%). Although the presence of the label of origin can be considered highly preferred in both cases (based on RPL model estimates, they are willing to pay about 1682 HUF for a product with the label of origin, compared to non-labeled product), the significant standard deviation value of the RPL model suggests that there is heterogeneity in willingness to pay for the attribute. In terms of place of purchase, we can draw similar conclusions in RPL as in the case of the MNL model (they prefer the farmers’ market over butcher and hyper-/supermarket and are willing to pay about 657 HUF less at a butcher, 1058 HUF less at a hyper-/supermarket purchase, compared to buying at the farmers’ market). However, it is important to note that we obtained a significant standard deviation value for the hyper-/supermarket, which suggests that there is heterogeneity in consumer willingness to pay for this level of attribute.

### 4.2. Consumer Segments of the Mangalica Sausage

Before presenting the results, it is important to note that several model configurations have been tested for the latent class model (Table 6). The best values came from the three-class specification shown in Table 7 (based on class probability values, Pseudo R^2^, Log-likelihood, and AIC aspects). 

It should also be noted that several sociodemographic variables were tested to find the source of heterogeneity among groups (members of groups with different characteristics, what preferences they have for each product feature). Among these, we found significant effects in terms of gender, age groups, and income levels (Table 7).

The parameter estimates clearly show that the coefficients of product attributes differ in size from the multinomial logit model, but similar conclusions can be drawn. The only significant difference is in Class 1 (“price sensitive”), where members prefer 75% mangalica meat content product more than 50% and 100% meat content (with a similar conclusion like in the RPL model). Based on significance values, all product attributes can be considered significant, except hyper-/supermarket level for Class 3 (“label neutral”).

It is important to note that for Class 1 (“price sensitive”), we found several significant sociodemographic variables, including gender, age 3, age 4, and income 4. Based on these, we can conclude that older (above 40 years) men with lower income level (average or below average) are most likely to be in Class 1 (“price sensitive”). They are significantly more price sensitive in case of the product investigated than the other two classes, preferring a product with a mangalica meat content of 75%, possessing the label of origin and obtaining the product from the ‘farmers’ market is very important to them.

The willingness to pay estimates for the model are shown in Table 8, where in addition to point estimates for each class, parameters for the entire model are indicated, corrected by class probability values. 

Based on the results of the WTP estimates (for the full model), we can conclude that consumers would pay about 993 HUF more for a 75% and 1165 HUF more for a 100% mangalica meat content product compared to 50% meat content product; pay a higher price of about 1897 HUF for a labeled product; and would give about 768 HUF less when purchasing at a butcher, while about 1143 HUF at a hyper-/supermarket shopping, compared to buying on ‘farmers’ market. Based on these results (shown in Table 7 and Table 8), we named the classes, which we have used earlier (price sensitive, loyal to label, and label neutral).

### 4.3. Changes in Consumer Willingness to Pay for a Traditional Mangalica Product 

To answer our last question, we compared the results of the 2012 and 2019 WTP estimates (shown in Table 9).

## 5. Discussion

In our paper, we tried to investigate the consumers’ preferences toward mangalica sausages, in terms of utilities and willingness to pay, and we also tried to compare our results to previous research of 2012. Regarding the willingness to pay, it can be concluded that while in the case of the MNL model, consumers would pay more for the 100% meat content product than for the 75% product (considering the 50% product as a base). In contrast, in the case of the RPL model, they would pay the most for the 75% meat content product. The fit of the applied LC model (based on the Pseudo R^2^ value) shows a better fit than the RPL model. Similar conclusions can be drawn for classes as for the MNL and RPL models. However, in terms of meat content, we can see contradictory results here as well (in the case of two classes, the product with 100% meat content is the most preferred, while in the case of one class, the product with 75% meat content).

Our results suggest that for this traditional Hungarian pork, there is heterogeneity in consumer preferences, in which the conclusion is similar to inferences of Font-i-Furnols and Guerrero [56] and Kallas, Čandek-Potokar, Tomažin, Pugliese, Aquilani, and Gil [59]. Our assumption that the changes in preferences are mainly related to the food labels and their information content can be confirmed, in line with other consumer studies focusing on traditional meat products (e.g., [82,83,84]). Comparing the willingness to pay for different product characteristics with the 2012 results, we can conclude that consumers would pay significantly more for both labeled and high-meat content products. They prefer to buy at the farmers’ market, though, in the 2012 survey [62], sourcing at the butcher was the most preferred place of purchase (for a detailed comparison, see Table 9). 

Moreover, the influence of consumers’ sociodemographic characteristics was also confirmed and supported by the results as older men with lower income represented a distinct class. Previous research including Van Loo, Caputo, Nayga, Meullenet, and Ricke [34], Wang, Ge, and Ma [35], Kallas et al. [60], Lusk [85], Lusk et al. [86], Verbeke et al. [87] reached a similar consequence. Based on their conclusions, product labeling is a determining factor in decisions, and they also shed light on the impact of sociodemographic characteristics in decision-making.

Regarding price levels and willingness to pay, a remarkable premium was identified. Our survey was conducted in the region (Northern Great Plain) where mangalica production is traditionally the most important in Hungary; therefore these local consumers prefer traditional meat products, and also have a higher willingness to pay, just as found for local consumers by van Zyl, Vermeulen, and Kirsten [36]. On the contrary, unlike another central European investigation found (Špička, Náglová, and Mezera [55] for the Czech Republic), for Hungarian mangalica pork meat consumers, sourcing from the butcher was preferred over purchasing in large scale food retails. This difference could be explained by the fact that the subject of the Czech research was conventional pork meat. This also suggests that for traditional food products, traditional sales channels are preferred and in Hungary, the farmers’ market is still considered as one of the most important channels [88], and with other types of short food supply chains are expected to have increasing importance in the case of quality food purchase [89].

For traditional pork products, Candek-Potokar, Giusto, Conti, Cosola, and Fontanesi [61] suggested—among others—a trademark which attracts both farmers, breeders’ associations, and processors; therefore, it could contribute to the self-sustainability of comprised local pig breeds. Our results show, that in the case of mangalica, the label of the producers’ associations testifying genuine origin has already filled this gap and consumers are attracted to this label, both in terms of utility and price premium.

This study has some limitations and further research opportunities. First, due to limited funding, it was conducted only in one region of Hungary. This study could be repeated in a national survey. Through this, we could examine the attitude of the entire Hungarian population to the purchase of mangalica sausage and could gain even more evidence. Second, the preference assessment procedures based on a hypothetical situation do not take into account many of the factors that arise in real choices [90]. Besides, the graphical representation of cards in decision situations were weak, sketchy, and less realistic, as opposed to other experiments [85,91,92]. Finally, the structure of the corresponding model specification also involves several limitations and issues (assigning random parameters, determining the number of latent classes).

## 6. Conclusions

In our research, we aimed to explore consumer preferences for a labeled traditional product, the mangalica sausage, using a discrete choice experiment and comparing our results with the previous investigation in the same topic with a very similar methodology and with the same attributes [62].

Our study was conducted on a sample of 477 persons. Product attributes featured in the experiment included label of origin, meat content, product price, and place of purchase. We made our estimates using multinomial logit, latent class, and random parameter logit models. Based on our results, we concluded that the existence of the label of origin positively influences the consumers’ sense of utility; purchasing at the farmers’ market is more preferred over the butcher and even more over hyper-/supermarket. However, the role of the level of mangalica meat content was contradictory.

The overall results of our research have clear messages for the stakeholders of the value chain of this traditional pork variety. On the one hand, the label of the Hungarian National Association of Mangalica Breeders testifying genuine origin has an added value, as consumers acknowledge it. On the other hand, mangalica sausage producers should be encouraged to use direct sales channels to reach their target groups.

Regarding willingness to pay, higher mangalica meat content is accepted by the consumers with a remarkable price premium compared to sausages with a lower level of mangalica meat content. This indicates that the higher quality level of this traditional variety over the conventional pork meat is recognized. This could also stimulate the production of mangalica meat. In addition, the label of the producers’ association is accompanied by a higher price level, indicating that it is worthy of investing by the producers and of maintaining the testifying system through their association. Last but not least, the negative price premium to be paid for non-direct sales (outside of farmers’ market) also reveals the importance of short food supply chains for mangalica producers.

In our paper, as a reference point, we used the results of a similar survey conducted in 2012. Since then, a change in consumers’ attitude can be recognized. Although the same product characteristics are still considered the most important, the governmental promotion campaigns and the initiatives of the Hungarian National Association of Mangalica Breeders have met their expectations. Mangalica products are consumed and preferred even more over mainstream alternative products, in terms of willingness to pay. The demand of this traditional product has moved to even more authentic channels and farmers’ markets providing direct interactions between the producers and the final consumers seem to be the place where this traditional product can fulfil a real niche of the market and can attract a determinative number of consumers. According to our LC model results, three consumer segments were identified: “price sensitive, loyal to label, label neutral” due to the preference heterogeneity. Furthermore, we can conclude that older (above 40 years) men with lower income level (average or below average) are most likely to be in the “price sensitive” separate group according to habits of mangalica sausage. These characteristics of the mangalica meat consumers should be also bear in mind once targeting this traditional product.

However, further research might also be addressed on the preferences of other traditional food products to investigate whether consumers consider the existence of product labeling properties also important. Besides, it may be worthy of testing additional model specifications and of including additional sociodemographic variables to refine the estimates, using a nation-wide sample. 

## Figures and Tables

**Table 1:** Attributes, their levels, and their coding.

Attribute	Attribute Level	Coding
Price (HUF/kg) ^1,2^	1500 HUF	Continuous variable
	2000 HUF	
	2500 HUF	
	3000 HUF	
Mangalica meat content (from the total meat) (%)	50%	1
	75%	2
	100%	3
Label of origin (NAMB label of origin ^3^)	No	0
	Yes	1
Place of purchase	‘Farmers’ market	1
	Butcher	2
	Hyper-/supermarket	3

^1^ 1 EUR is 332 HUF based on the exchange rate of on 9 December 2019. ^2^ The levels of price attribute are divided by 1000 in order to get fewer ranges. ^3^ The National Association of Mangalica Breeders (NAMB) certifies that registered mangalica pig meat has been used in the preparation of the product.

**Table 2:** An example of a decision situation.

	Alternative 1	Alternative 2	Alternative 3
Price (HUF/kg)	3000	2000	None of these products
Meat content (%)	75%	75%	
Label of origin	Yes	No	
Place of purchase	‘Farmers’ market	Butcher	

**Table 3:** Sociodemographic characteristics of respondents.

Sociodemographic Factors	Sample (*N* = 477)	Regional Distribution *
Gender (%)		
Female	56.0	51.7
Male	44.0	48.3
Age (category) (%)		
Age1 (<30)	22.0	21.8
Age2 (30–39)	26.5	27.1
Age3 (40–49)	22.0	21.0
Age4 (50<)	29.5	30.1
Age (mean)	41.54	41.7
Highest level of education (%)		
Elementary	8.2	-
Secondary	44.6	-
Higher education	47.2	-
Monthly gross income (%) **		
Substantially below average	33.3	-
Below average	17.6	-
Average	25.8	-
Above average	23.3	-
Residence (%)		
Urban	72.3	68.3
Rural	27.7	31.7

* [66]; ** Net average regional income in 2019: 187,366 HUF/month.

**Table 4:** The results of multinomial logit (MNL) and random parameter logit (RPL) model estimates.

	MNL Model		RPL Model (Direct WTP)	
Attributes and Model Details	Coefficient	Standard Error	Coefficient	Standard Error
ASC (alternative 2)	0.652 ***	0.071	0.673 ***	0.061
ASC (opt-out)	−1.583 ***	0.138	−3.191 ***	0.156
Price/1000	−0.885 ***	0.058	−1.215 *** (2.909) ***	0.138
75% meat content	0.697 ***	0.078	0.895 ***	0.039
100% meat content	0.844 ***	0.065	0.862 ***	0.044
Label of origin	1.843 ***	0.089	1.682 *** (0.677) ***	0.073
Butcher	−0.759 ***	0.09	−0.657 ***	0.064
Hyper-/supermarket	−1.009 ***	0.101	−1.058 *** (0.585) ***	0.101
Observations	3816			
Pseudo R^2^	0.1608		0.2634	
Adj R^2^	0.1589		0.2607	
Log-likelihood	−3518.227		−3088.236	
AIC	7052.45		6198.47	

Note: ASC represents the alternative-specific constant value.; ASC (alternative 1), 50% meat content, no label of origin, and the ‘farmers’ market variables reported the base levels in the estimates.; The standard deviation values in the RPL model (for random variables) are shown in parentheses below the parameter estimates.; *** indicate statistical significance at the 1% level.; ASC (alternative 2), ASC (opt-out), and price coefficients in RPL model mean the coefficient of utility, while the others (75% meat content, 100% meat content, label of origin, butcher, hyper-/supermarket) mean the coefficients of willingness to pay (WTP).; Adj R^2^ denotes the adjusted value of R^2^; AIC denotes the Akaike information criterion.

**Table 5:** Willingness to pay (WTP) estimates for the multinomial logit model.

Product Attributes	Willingness to Pay
75% meat content	0.787 ***
100% meat content	0.954 ***
Label of origin	2.082 ***
Butcher	−0.858 ***
Hyper-/supermarket	−1.139 ***

Note: *** indicate statistical significance at the 1% level.

**Table 6:** Comparison of information criteria.

	2 Segments Model		3 Segments Model			4 Segments Model			
Estimated parameters	22		36			50			
Log-likelihood (LL)	−3092.223		−2993.281			−2943.919			
AIC	6228.45		6058.56			5987.84			
BIC	6365.88		6283.45			6300.19			
Pseudo R^2^	0.2624		0.286			0.2978			
Class probability values	0.40	0.60	0.28	0.57	0.15	0.13	0.54	0.04	0.29

Note: Log-likelihood evaluated at zero is: −4192.304.; AIC denotes the Akaike information criterion, while BIC denotes the Bayesian information criterion.

**Table 7:** The results of the latent class (LC) model estimates.

Attributes and Model Details	Coefficient			Standard Error		
	Price Sensitive	Loyal to Label	Label Neutral	Price Sensitive	Loyal to Label	Label Neutral
ASC (alternative 2)	0.62 ***			0.088		
ASC (opt-out)	−2.845 ***			0.184		
Price/1000	−3.663 ***	−0.55 ***	−1.915 ***	0.247	0.099	0.129
75% meat content	3.457 ***	0.584 ***	1.53 ***	0.315	0.142	0.256
100% meat content	3.07 ***	0.73 ***	2.223 ***	0.341	0.112	0.265
Label of origin	6.98 ***	1.26 ***	0.722 ***	0.388	0.174	0.255
Butcher	−2.214 ***	−0.524 ***	−0.714 ***	0.25	0.167	0.228
Hyper-/supermarket	−2.478 ***	−0.711 ***	−2.783	0.257	0.145	0.00
Female	−0.859 ***		−0.029	0.24		0.35
Age2	0.00		0.153	0.357		0.494
Age3	1.141 ***		1.073 **	0.355		0.46
Age4	0.771 **		0.668	0.327		0.47
Income2	0.203		1.573 ***	0.35		0.45
Income3	−0.035		−0.025	0.285		0.481
Income4	−0.986 ***		−0.279	0.329		0.457
Delta	−0.558		−2.112 ***	0.313		0.539
Class probability values	0.28	0.57	0.15			
Observations	3816					
Pseudo R^2^	0.286					
Adj R^2^	0.2774					
Log-likelihood	−2993.281					
AIC	6058.56					

Note: ASC represents the alternative-specific constant value.; Female: type of gender, Age 2 (30–40 years), Age 3 (40–50 years), Age 4 (above 50 year) the age, Income 2 (below average), Income 3 (average), and Income 4 (above average) represent the monthly gross income classification for respondents.; ASC (alternative 1), 50% meat content, no label of origin, ‘farmers’ market, male, the lowest age group (below 30 years) and income level (substantially below average), and the delta variable for class “B” reported the base levels in the estimates.; Delta is a constant value for the classes of the latent class model.; **, and *** indicate statistical significance at the 5% and 1% levels.

**Table 8:** WTP estimates for the LC model.

Product Attributes	Willingness to Pay			
	Price Sensitive	Loyal to Label	Label Neutral	Full Model
75% mangalica meat content	0.944 ***	1.061 ***	0.799 ***	0.993 ***
100% mangalica meat content	0.838 ***	1.326 ***	1.161 ***	1.165 ***
Label of origin	1.906 ***	2.289 ***	0.377 **	1.897 ***
Butcher	−0.604 ***	−0.952 **	−0.373 **	−0.768 **
Hyper-/supermarket	−0.677 ***	−1.291 ***	−1.453 ***	−1.143 ***

Note: **, and *** indicate statistical significance at the 5% and 1% levels.

**Table 9:** Comparisons of WTP estimates for MNL and RPL models between 2012 and 2019.

Product Attributes	WTP for MNL (HUF)		WTP for RPL (HUF)	
	2012	2019	2012	2019
Label of origin	0.457	2.082	0.942	1.682
75% meat content	0.235	0.787	0.623	0.895
100% meat content	0.445	0.954	0.736	0.862
Butcher	0.349	−0.858	0.827	−0.657
Hyper-/supermarket	−0.715	−1.139	−1.347	−1.058

## Appendix A

In the multinomial logit model, the systematic part of the utility for alternative *i* can be written according to Equation (A1):

(A1) $$\begin{array}{ll} V_{i}= & ASC_{alt.i}+\beta_{Price}*Price_{alt.i}+\beta_{75\%\;Meat\;content}*75\%\;Meat\;content_{alt.i}\\ & +\beta_{100\%\;Meat\;content}*100\%\;Meat\;content_{alt.i}+\beta_{Label\;of\;origin}\\ & *Label\;of\;origin_{alt.i}+\beta_{Butcher}*Butcher_{alt.i}\\ & +\beta_{Hyper-/supermarket}*Hyper_{alt.i}. \end{array}$$

The systematic part of the utility in the RPL model can be written according to Equation (A2):

(A2) $$\begin{array}{ll} V_{i}= & ASC_{alt.i}+\beta_{Price}*(Price_{alt.i}+V_{75\%\;Meat\;content}*75\%\;Meat\;content_{alt.i}\\ & +V_{100\%\;Meat\;content}*100\%\;Meat\;content_{alt.i}+V_{Label\;of\;origin}\\ & *Label\;of\;origin_{alt.i}+V_{Butcher}*Butcher_{alt.i}\\ & +V_{Hyper-/supermarket}*Hyper_{alt.i}), \end{array}$$

where the *V* parameters represent the willingness to pay for that attribute.

The willingness to pay calculation in the MNL model is based on Equation (A3):

(A3) $$WTP_{attribute\;k}=(-)\frac{\beta_{attribute\;k}}{\beta_{attribute\;Price}},$$

where *β* expresses the value of the coefficients for attributes.

For the latent class model, the systematic part of utility for alternative *i* and class *q* can be written according to Equation (A4):

(A4) $$\begin{array}{ll} V_{i}= & ASC_{alt.i}+\beta_{Price}\left[q\right]*Price_{alt.i}+\beta_{75\%\;Meat\;content}\left[q\right]\\ & *75\%\;Meat\;content_{alt.i}+\beta_{100\%\;Meat\;content}\left[q\right]\\ & *100\%\;Meat\;content_{alt.i}+\beta_{Label\;of\;origin}\left[q\right]\\ & *Label\;of\;origin_{alt.i}+\beta_{Butcher}\left[q\right]*Butcher_{alt.i}\\ & +\beta_{Hyper-/supermarket}\left[q\right]*Hyper_{alt.i}. \end{array}$$

## References

[1] Novemsky N., Dhar R., Schwarz N., Simonson I. (2007). Preference Fluency in Choice. J. Mark. Res..

[2] Kumar N., Kapoor S. (2017). Do labels influence purchase decisions of food products? Study of young consumers of an emerging market. Br. Food J..

[3] Szakály Z., Horvát A., Soós M., Pető K., Szente V. (2014). A minőségre és származásra utaló jelölések szerepe a fogyasztói döntéshozatalban. Élelmiszertáplálkozás Mark..

[4] Szakály Z., Soós M., Szabó S., Szente V. (2016). Role of labels referring to quality and country of origin in food consumers’ decisions. Acta Aliment..

[5] The United Nations The Division for Sustainable Development Goals (DSDG). https://sustainabledevelopment.un.org/.

[6] Guerrero L., Claret A., Verbeke W., Enderli G., Zakowska-Biemans S., Vanhonacker F., Issanchou S., Sajdakowska M., Granli B.S., Scalvedi L. (2010). Perception of traditional food products in six European regions using free word association. Food Qual. Prefer..

[7] Kühne B., Vanhonacker F., Gellynck X., Verbeke W. (2010). Innovation in traditional food products in Europe: Do sector innovation activities match consumers’ acceptance?. Food Qual. Prefer..

[8] Pieniak Z., Verbeke W., Vanhonacker F., Guerrero L., Hersleth M. (2009). Association between traditional food consumption and motives for food choice in six European countries. Appetite.

[9] Chrysochou P., Krystallis A., Giraud G. (2012). Quality assurance labels as drivers of customer loyalty in the case of traditional food products. Food Qual. Prefer..

[10] Török Á. (2011). Spanyolul tanul a magyar mangalica!. Gazdálkodás.

[11] Balogh P., Szabó P., Pocsai K. (2013). Introduction of different mangalica breeds’s prolificacy and rearing performances. Res. Pig Breed..

[12] National Association of Mangalica Breeders. Introduction. http://www.moe.org.hu/en/association/introduction/.

[13] National Association of Mangalica Breeders. https://magyarmezogazdasag.hu/cimkek/mangalicatenyesztok-orszagos-egyesulete-moe.

[14] Simon H.A. (1955). A Behavioral Model of Rational Choice. Q. J. Econ..

[15] Simon H.A. (1986). Rationality in Psychology and Economics. J. Bus..

[16] Kroneberg C., Kalter F. (2012). Rational Choice Theory and Empirical Research: Methodological and Theoretical Contributions in Europe. Annu. Rev. Sociol..

[17] McFadden D. (2001). Economic Choices. Am. Econ. Rev..

[18] Hess S., Daly A. (2014). Handbook of Choice Modelling.

[19] Hensher D.A., Rose J.M., Greene W.H. (2005). Applied Choice Analysis.

[20] Georgescu I. (2007). Fuzzy choice functions. Fuzzy Choice Functions.

[21] Kroes E.P., Sheldon R.J. (1988). Stated preference methods: An introduction. J. Transp. Econ. Policy.

[22] Mark T.L., Swait J. (2004). Using stated preference and revealed preference modeling to evaluate prescribing decisions. Health Econ..

[23] Hensher D.A., Barnard P.O., Truong T.P. (1988). The role of stated preference methods in studies of travel choice. J. Transp. Econ. Policy.

[24] McCluskey J.J., Huffman W.E. (2017). Using Stated Preference Techniques and Experimental Auction Methods: A Review of Advantages and Disadvantages for Each Method in Examining Consumer Preferences for New Technology. Int. Rev. Environ. Resour. Econ..

[25] Hensher D.A., Bradley M. (1993). Using stated response choice data to enrich revealed preference discrete choice models. Mark. Lett..

[26] Train K., Wilson W.W. (2008). Estimation on stated-preference experiments constructed from revealed-preference choices. Transp. Res. Part B Methodol..

[27] Costanigro M., Onozaka Y. (2019). A Belief-Preference Model of Choice for Experience and Credence Goods. J. Agric. Econ..

[28] Louviere J.J., Hensher D.A., Swait J.D. (2000). Stated Choice Methods: Analysis and Applications.

[29] Loureiro M.L., McCluskey J.J. (2000). Assessing consumer response to protected geographical identification labeling. Agribusiness.

[30] Lancaster K.J. (1966). A New Approach to Consumer Theory. J. Political Econ..

[31] Ceschi S., Canavari M., Castellini A. (2017). Consumer’s Preference and Willingness to Pay for Apple Attributes: A Choice Experiment in Large Retail Outlets in Bologna (Italy). J. Int. Food Agribus. Mark..

[32] Denver S., Jensen J.D. (2014). Consumer preferences for organically and locally produced apples. Food Qual. Prefer..

[33] Lockshin L., Jarvis W., d’Hauteville F., Perrouty J.-P. (2006). Using simulations from discrete choice experiments to measure consumer sensitivity to brand, region, price, and awards in wine choice. Food Qual. Prefer..

[34] Van Loo E.J., Caputo V., Nayga R.M., Meullenet J.-F., Ricke S.C. (2011). Consumers’ willingness to pay for organic chicken breast: Evidence from choice experiment. Food Qual. Prefer..

[35] Wang J., Ge J., Ma Y. (2018). Urban Chinese Consumers’ Willingness to Pay for Pork with Certified Labels: A Discrete Choice Experiment. Sustainability.

[36] van Zyl K., Vermeulen H., Kirsten J.F. (2013). Determining South African consumers’ willingness to pay for certified Karoo lamb: An application of an experimental auction. Agrekon.

[37] AND International (2012). Value of Production of Agricultural Products and Foodstuffs, Wines, Aromatised Wines and Spirits Protected by a Geographical Indication (GI).

[38] Ardeshiri A., Rose J.M. (2018). How Australian consumers value intrinsic and extrinsic attributes of beef products. Food Qual. Prefer..

[39] Gao Z., Schroeder T.C., Yu X. (2010). Consumer Willingness to Pay for Cue Attribute: The Value Beyond Its Own. J. Int. Food Agribus. Mark..

[40] Loureiro M.L., Umberger W.J. (2003). Estimating consumer willingness to pay for country-of-origin labeling. J. Agric. Resour. Econ..

[41] Revoredo-Giha C., Lamprinopoulou C., Leat P., Kupiec-Teahan B., Toma L., Cacciolatti L. (2011). How Differentiated Is Scottish Beef? An Analysis of Supermarket Data. J. Food Prod. Mark..

[42] Arnoult M., Lobb A., Tiffin R. (2010). Willingness to Pay for Imported and Seasonal Foods: A UK Survey. J. Int. Food Agribus. Mark..

[43] Bernabeu R., Rabadan A., El Orche N.E., Diaz M. (2018). Influence of quality labels on the formation of preferences of lamb meat consumers. A Spanish case study. Meat Sci.

[44] Gracia A. (2013). Consumers’ preferences for a local food product: A real choice experiment. Empir. Econ..

[45] Gracia A., de Magistris T., Nayga R.M. (2012). Importance of Social Influence in Consumers’ Willingness to Pay for Local Food: Are There Gender Differences?. Agribusiness.

[46] Imami D., Chan-Halbrendt C., Zhang Q., Zhllima E. (2011). Conjoint analysis of consumer preferences for lamb meat in central and southwest urban Albania. Int. Food Agribus. Manag. Rev..

[47] Arfini F., Mancini M.C. (2015). The effect of information and co-branding strategies on consumers willingness to pay (WTP) for Protected Designation of Origin (PDO) products: The case of pre-sliced Parma Ham. Prog. Nutr..

[48] Mesías F., Gaspar P., Escribano M., Pulido F. (2010). The role of protected designation of origin in consumer preference for Iberian dry-cured ham in Spain. Ital. J. Food Sci..

[49] Resano H., Sanjuán A.I., Albisu L.M. (2012). Consumers’ response to the EU Quality policy allowing for heterogeneous preferences. Food Policy.

[50] Garavaglia C., Mariani P. (2017). How Much Do Consumers Value Protected Designation of Origin Certifications? Estimates of willingness to Pay for PDO Dry-Cured Ham in Italy. Agribusiness.

[51] Balcombe K., Bradley D., Fraser I., Hussein M. (2016). Consumer preferences regarding country of origin for multiple meat products. Food Policy.

[52] Shan L.C., De Brún A., Henchion M., Li C., Murrin C., Wall P.G., Monahan F.J. (2017). Consumer evaluations of processed meat products reformulated to be healthier–A conjoint analysis study. Meat Sci..

[53] Di Vita G., Blanc S., Mancuso T., Massaglia S., La Via G., D’Amico M. (2019). Harmful Compounds and Willingness to Buy for Reduced-Additives Salami. An Outlook on Italian Consumers. Int. J. Environ. Res. Public Health.

[54] Ngapo T. (2017). Consumer preferences for pork chops in five Canadian provinces. Meat Sci..

[55] Špička J., Náglová Z., Mezera J. Consumers Behaviour in the Czech Pork Meat Retail Market. Proceedings of the INPROFORUM 2017.

[56] Font-i-Furnols M., Guerrero L. (2014). Consumer preference, behavior and perception about meat and meat products: An overview. Meat Sci..

[57] Xazela N.M., Hugo A., Marume U., Muchenje V. (2017). Perceptions of rural consumers on the aspects of meat quality and health implications associated with meat consumption. Sustainability.

[58] Merlino V.M., Borra D., Verduna T., Massaglia S. (2017). Household behavior with respect to meat consumption: Differences between households with and without children. Vet. Sci..

[59] Kallas Z., Čandek-Potokar M., Tomažin U., Pugliese C., Aquilani C., Gil J.M. (2017). Measuring consumers’ preferences for traditional and innovative pork products. Agric. Conspec. Sci..

[60] Kallas Z., Varela E., Čandek-Potokar M., Pugliese C., Cerjak M., Tomažin U., Karolyi D., Aquilani C., Vitale M., Gil J.M. (2019). Can innovations in traditional pork products help thriving EU untapped pig breeds? A non-hypothetical discrete choice experiment with hedonic evaluation. Meat Sci..

[61] Candek-Potokar M., Giusto A., Conti C., Cosola C., Fontanesi L. (2018). Improving sustainability of local pig breeds using quality labels–case review and trademark development in project TREASURE. Arch. Zootec..

[62] Balogh P., Békési D., Gorton M., Popp J., Lengyel P. (2016). Consumer willingness to pay for traditional food products. Food Policy.

[63] AMC Kampány Indult a Mangalicahús Népszerűsítéséért. https://www.amc.hu/belpiaci-hirek/kampany-indult-a-mangalicahus-nepszerusiteseert/576/.

[64] Ngene C. (2018). 1.2 User Manual & Reference Guide.

[65] Hungarian Central Statistical Office National Data—Summary Tables. http://www.ksh.hu/stadat.

[66] Hungarian Central Statistical Office National Data—Dissemination Database. http://statinfo.ksh.hu/Statinfo/themeSelector.jsp?lang=hu.

[67] Hess S., Palma D. (2019). Apollo: A flexible, powerful and customisable freeware package for choice model estimation and application. J. Choice Model..

[68] Hess S., Palma D. Apollo Version 0.0.6, User Manual. www.ApolloChoiceModelling.com.

[69] McFadden D., Zarembka P., Zarembka P. (1973). Frontiers in econometrics. Cond. Logit Anal. Qual. Choice Behav. Frontiers in Econometrics.

[70] Fiebig D.G., Keane M.P., Louviere J., Wasi N. (2010). The Generalized Multinomial Logit Model: Accounting for Scale and Coefficient Heterogeneity. Mark. Sci..

[71] Fosgerau M., Bierlaire M. (2007). A practical test for the choice of mixing distribution in discrete choice models. Transp. Res. Part B Methodol..

[72] Train K.E. (2003). Discrete Choice Methods with Simulation.

[73] Train K., Weeks M., Scarpa R., Alberini A. (2005). Discrete Choice Models in Preference Space and Willingness-to-Pay Space. Applications of Simulation Methods in Environmental and Resource Economics.

[74] Hensher D.A., Rose J.M., Greene W.H. (2008). Combining RP and SP data: Biases in using the nested logit ‘-contrasts with flexible mixed logit incorporating panel and scale effects. J. Transp. Geogr..

[75] Boxall P.C., Adamowicz W.L. (2002). Understanding heterogeneous preferences in random utility models: A latent class approach. Environ. Resour. Econ..

[76] Greene W.H., Hensher D.A. (2003). A latent class model for discrete choice analysis: Contrasts with mixed logit. Transp. Res. Part B Methodol..

[77] Morey E., Thacher J., Breffle W. (2006). Using Angler Characteristics and Attitudinal Data to Identify Environmental Preference Classes: A Latent-Class Model. Environ. Resour. Econ..

[78] Cavanaugh J.E., Neath A.A. (2019). The Akaike information criterion: Background, derivation, properties, application, interpretation, and refinements. Wiley Interdiscip. Rev. Comput. Stat..

[79] Ballco P., De Magistris T. (2019). Spanish Consumer Purchase Behaviour and Stated Preferences for Yoghurts with Nutritional and Health Claims. Nutrients.

[80] Jurado F., Gracia A. (2017). Does the valuation of nutritional claims differ among consumers? Insights from Spain. Nutrients.

[81] Hess S., Daly A., Dekker T., Cabral M.O., Batley R. (2017). A framework for capturing heterogeneity, heteroskedasticity, non-linearity, reference dependence and design artefacts in value of time research. Transp. Res. Part B Methodol..

[82] Bernués A., Olaizola A., Corcoran K. (2003). Labelling information demanded by European consumers and relationships with purchasing motives, quality and safety of meat. Meat Sci..

[83] Bernués A., Ripoll G., Panea B. (2012). Consumer segmentation based on convenience orientation and attitudes towards quality attributes of lamb meat. Food Qual. Prefer..

[84] Cerjak M., Karolyi D., Kovačić D. (2011). Effect of information about pig breed on consumers’ acceptability of dry sausage. J. Sens. Stud..

[85] Lusk J.L. (2018). Consumer preferences for and beliefs about slow growth chicken. Poult. Sci..

[86] Lusk J.L., Schroeder T.C., Tonsor G.T. (2013). Distinguishing beliefs from preferences in food choice. Eur. Rev. Agric. Econ..

[87] Verbeke W., Guerrero L., Almli V.L., Vanhonacker F., Hersleth M. (2016). European Consumers’ Definition and Perception of Traditional Foods. Traditional Foods.

[88] Vittersø G., Torjusen H., Laitala K., Tocco B., Biasini B., Csillag P., de Labarre M.D., Lecoeur J.-L., Maj A., Majewski E. (2019). Short Food Supply Chains and Their Contributions to Sustainability: Participants’ Views and Perceptions from 12 European Cases. Sustainability.

[89] Delicato C., Collison M., Myronyuk I., Symochko T., Boyko N. (2019). Is Local Better? Consumer Value in Food Purchasing and the Role of Short Food Supply Chains. Stud. Agric. Econ..

[90] Lopéz-Galán B., de-Magistris T. (2020). Personal and Psychological Traits Influencing the Willingness to Pay for Food with Nutritional Claims: A Comparison between Vice and Virtue Food Products. Foods.

[91] Grashuis J., Magnier A. (2018). Product differentiation by marketing and processing cooperatives: A choice experiment with cheese and cereal products. Agribusiness.

[92] Profeta A., Hamm U. (2019). Do consumers prefer local animal products produced with local feed? Results from a Discrete-Choice experiment. Food Qual. Prefer..

